# The importance of individualized article-specific metrics for evaluating research productivity

**DOI:** 10.1186/1742-4690-6-82

**Published:** 2009-09-16

**Authors:** Kuan-Teh Jeang

**Affiliations:** 1 National Institutes of Health, Bethesda, MD, USA

## Abstract

This editorial discusses the rationale for using article-specific rather than journal-specific metrics for evaluating highly published authors.

## Editorial

Mark Patterson of PLoS (Public Library of Science) recently wrote an online piece  on how to measure impact where it matters. Patterson makes an important point that one should focus on article specific metrics when evaluating a published paper rather than relying "on the name and the impact factor (IF) of the journal in which the work is published". In the past, it was not always easy to assess quickly and accurately the citations to individually published articles. Today, many electronic tools (e.g. ISI Web of Science, Scopus, Google Scholar) exist that can accomplish this task facilely and reliably. Because there are inherent shortcomings to how a journal's IF is calculated and because of the rather poor representativeness of the IF for the citations to individual articles [1,2], institutions and peer-review bodies should be encouraged strongly to employ article-specific measures in preference to journal IFs in evaluations.

Article-specific citations are often not used properly in evaluating published authors. For example, in some circles, it has become fashionable to create lists of "highly cited" scientists in various fields (e.g. ; highly cited in immunology, highly cited in microbiology, highly cited in molecular biology and genetics etc...). In some respects, these lists could be useful conveniences, provided that the users understand clearly how they are generated and what they mean (and do not mean). One could assume that "highly cited in microbiology" is based on article specific-citations. In fact, this would be a mistaken assumption because the listing is actually based on journal-specific data. What does this mean? By way of explanation, let's consider a hypothetical illustration. If John Smith were an author of 10 papers on HIV-1 published in *Cell *or the *Journal of Biological Chemistry *(which are not counted by ISIHighlyCited as microbiology journals) and if these 10 papers were cited cumulatively 1,000 times over a specified duration, then Smith's citation counts based on these papers for purposes of "highly cited in microbiology" would be 0. On the other hand, if the exactly same 10 Smith papers on HIV were unsuccessful in initial submissions to *Cell *or the *Journal of Biological Chemistry*, but were subsequently successfully published in the *Journal of Virology*, *Retrovirology*, or *Virology *(all counted as microbiology journals), then the 1,000 citations to these papers would add 1,000 counts to Smith's ranking for purposes of "highly cited in microbiology". So, here is an example where journal-specific metrics trump article-specific measures. In order to be "highly cited in microbiology", what one publishes (i.e. article-specific content on HIV) counts not unless it is published in a journal deemed as "microbiology" (i.e. a journal-specific metric). Thus, this illustration shows that ratings based on journal-specific data that do not properly integrate article-specific measures can be misleading when used to rate scientists. For retrovirologists, *Retrovirology *has emphasized consistently the use of person-specific measures of H-index [3] and total citations. Indeed, annually for the past three years, these data have been presented, using the Scopus data base , for selected *Retrovirology *editorial board members (see Table 1) [1,2].

**Table 1:** H-index and citation frequencies of selected Retrovirology editorial board members.

**Title**	**Name**	**Role within *Retrovirology***	**Institution**	**City**	**Country**	**H index**	**Total times cited since 1996**
Dr.	Kuan-Teh Jeang	Editor-in-Chief	NIH	Bethesda	USA	46	9799

Dr.	Monsef Benkirane	Editor	CNRS	Montpellier	France	23	2210

Dr.	Ben Berkhout	Editor	Academic Med. Ctr	Amsterdam	the Netherlands	40	6925

Dr.	Andrew Lever	Editor	Cambridge University	Cambridge	UK	19	2065

Dr.	Mark Wainberg	Editor	McGill University	Montreal	Canada	40	10058

Dr.	Masahiro Fujii	Editor	Niigata University	Niigata	Japan	21	2186

Dr.	Michael Lairmore	Editor	Ohio State University	Columbus	USA	21	2226

Dr.	Michael Bukrinsky	Ed Board	George Washington Univ	Washington DC	USA	26	5218

Dr.	Dong-yan Jin	Ed Board	Hong Kong U	Hong Kong	China	25	2675

Dr.	Klaus Strebel	Ed Board	NIH	Bethesda	USA	27	4395

Dr.	Tom J. Hope	Ed Board	U. Illinois	Chicago	USA	27	4730

Dr.	Stephane Emiliani	Ed Board	Cochin Institute	Paris	France	19	2061

Dr.	Patrick Green	Ed Board	Ohio State University	Columbus	USA	19	1050

Dr.	Mauro Giacca	Ed Board	Int. Ctr. Genetics	Trieste	Italy	38	5795

Dr.	Olivier Schwartz	Ed Board	Institut Pasteur	Paris	France	31	5209

Dr.	Leonid Margolis	Ed Board	National Inst Child Health	Bethesda	USA	23	2028

Dr.	Fatah Kashanchi	Ed Board	George Washington U.	Washington DC	USA	27	2725

Dr.	Masao Matsuoka	Ed Board	Kyoto University	Kyoto	Japan	29	3834

Dr.	Naoki Mori	Ed Board	University of the Ryukyus	Okinawa	Japan	28	3375

Dr.	Chou-Zen Giam	Ed Board	Uniform Services Med School	Bethesda	USA	16	1698

Dr.	David Derse	Ed Board	NCI	Frederick	USA	15	1828

Dr.	Tatsuo Shioda	Ed Board	Osaka Univ	Osaka	Japan	24	2110

Dr.	John Semmes	Ed Board	Eastern Virginia Med College	Norfolk	USA	29	3416

Dr.	Anne Gatignol	Ed Board	McGill Univ.	Montreal	Canada	17	1542

Dr.	Rogier Sanders	Ed Board	Academic Med. Ctr	Amsterdam	the Netherlands	13	955

Dr.	Chen Liang	Ed Board	McGill Univ.	Montreal	Canada	19	976

Dr.	Finn Skou Pedersen	Ed Board	University of Aarhus	Aarhus	Denmark	19	1498

Dr.	Renaud Mahieux	Ed Board	Pasteur Int.	Paris	France	24	1489

Dr.	Neil Almond	Ed Board	NIBSC	Potters Bar	UK	15	1370

Dr.	Stephen P. Goff	Ed Board	Columbia University	New York	USA	44	14851

Dr.	Johnson Mak	Ed Board	Burnet Inst. Med. Research	Victoria	Australia	17	1679

Dr.	Christine Kozak	Ed Board	NIH	Bethesda	USA	29	7814

Dr.	Greg Towers	Ed Board	Univ. College	London	UK	17	1558

Dr.	Eric Cohen	Ed Board	Univ. Montreal	Montreal	Canada	37	7047

Dr.	Warner Greene	Ed Board	UCSF	San Francisco	USA	42	11011

Dr.	Jean-luc Darlix	Ed Board	U. Lyon	Lyon	France	33	6070

Dr.	Eric Freed	Ed Board	NCI	Frederick	USA	31	4906

Dr.	Toshiki Watanabe	Ed Board	Univ. of Tokyo	Tokyo	Japan	24	2576

Dr.	Mari Kannagi	Ed Board	Tokyo Med and Dental U	Tokyo	Japan	17	1474

Dr.	Frank Kirchhoff	Ed Board	University of Ulm	Ulm	Germany	34	5478

Dr.	Jennifer Nyborg	Ed Board	Colorado State U	Fort Collins	USA	18	1671

Dr.	Akifumi Takaori-Kondo	Ed Board	Kyoto University	Kyoto	Japan	14	718

Dr.	Marc Sitbon	Ed Board	CNRS	Montpellier	France	13	814

Dr.	Paul Gorry	Ed Board	MacFarlane Burnet Institute	Melbourne	Australia	16	835

Dr.	David Harrich	Ed Board	Queensland Inst Medical Res.	Brisbane	Australia	12	1063

Dr.	Susan Marriott	Ed Board	Baylor	Houston	USA	15	1102

Dr.	Alan Cochrane	Ed Board	U Toronto	Toronto	Canada	11	1191

Dr.	Yiming Shao	Ed Board	China CDC	Beijing	China	14	1123

Dr.	Vinayaka Prasad	Ed Board	Albert Einstein College Medicine	New York	USA	19	1239

Dr.	Roger Pomerantz	Ed Board	Tibotec	Yardley	USA	34	6912

Dr.	Li Wu	Ed Board	Medical College Wisconsin	Milwaukee	USA	30	5617

Dr.	Anne-Mieke Vandamme	Ed Board	Rega Inst. and Univ Hospitals	Leuven	Belgium	35	4994

Dr.	Alan Engelman	Ed Board	Harvard Univ.	Boston	USA	25	4070

Dr.	Paul Clapham	Ed Board	Univ. Massachusetts	Worcester	USA	30	6495

Dr.	Vinay Pathak	Ed Board	NCI	Frederick	USA	25	1951

Dr.	Jeremy Luban	Ed Board	Univ. Geneva	Geneva	Switzerland	29	4469

Finally, one should not overlook the merits of awards and prizes in evaluating highly accomplished colleagues. Awards/prizes can come in two flavors; one as "leading" and the other as "lagging" indicators of scientific potential/productivity. For example, "life-time achievement" awards would be a "lagging" measure of one's achievements, while a "young" investigator prize might be a "leading" indicator of future potential. *Retrovirology *annually awards a "*Retrovirology Prize*" to a mid-career scientist [4,5]. The Prize aims to recognize "lagging" and "leading" benchmarks. It rewards the past achievements of a scientist who is in his/her mid-career and who still has substantial lead-time to accomplish future breakthrough research in retrovirology [5-8]. With this editorial, this year's nomination period for the 2009 *Retrovirology Prize *to recognize a retrovirologist for non-HIV-retrovirology research is open. The nomination period will close on October 31, 2009. The rules for nomination and the selection procedures remain the same as in past years [9,10]. Interested individuals can direct email inquiries to editorial@retrovirology.com.

## Authors' contributions

KTJ wrote this editorial.
